# Where do we Stand after Decades of Studying Human Cytomegalovirus?

**DOI:** 10.3390/microorganisms8050685

**Published:** 2020-05-08

**Authors:** Francesca Gugliesi, Alessandra Coscia, Gloria Griffante, Ganna Galitska, Selina Pasquero, Camilla Albano, Matteo Biolatti

**Affiliations:** 1 Laboratory of Pathogenesis of Viral Infections, Department of Public Health and Pediatric Sciences, University of Turin, 10126 Turin, Italy; francesca.gugliesi@unito.it (F.G.); gloria.griffante@unito.it (G.G.); ganna.galitska@unito.it (G.G.); selina.pasquero@unito.it (S.P.); camilla.albano@unito.it (C.A.); 2 Complex Structure Neonatology Unit, Department of Public Health and Pediatric Sciences, University of Turin, 10126 Turin, Italy; alessandra.coscia@unito.it

**Keywords:** human cytomegalovirus, genetic variability, viral dissemination, pathogenesis, antiviral therapy

## Abstract

Human cytomegalovirus (HCMV), a linear double-stranded DNA betaherpesvirus belonging to the family of Herpesviridae, is characterized by widespread seroprevalence, ranging between 56% and 94%, strictly dependent on the socioeconomic background of the country being considered. Typically, HCMV causes asymptomatic infection in the immunocompetent population, while in immunocompromised individuals or when transmitted vertically from the mother to the fetus it leads to systemic disease with severe complications and high mortality rate. Following primary infection, HCMV establishes a state of latency primarily in myeloid cells, from which it can be reactivated by various inflammatory stimuli. Several studies have shown that HCMV, despite being a DNA virus, is highly prone to genetic variability that strongly influences its replication and dissemination rates as well as cellular tropism. In this scenario, the few currently available drugs for the treatment of HCMV infections are characterized by high toxicity, poor oral bioavailability, and emerging resistance. Here, we review past and current literature that has greatly advanced our understanding of the biology and genetics of HCMV, stressing the urgent need for innovative and safe anti-HCMV therapies and effective vaccines to treat and prevent HCMV infections, particularly in vulnerable populations.

## 1. Introduction

Human cytomegalovirus (HCMV), also called human herpesvirus 5 (HHV-5), is one of the nine herpesviruses capable of successfully infecting humans. HCMV belongs to the Group I of the Baltimore classification, and specifically to the subfamily *Betaherpesvirinae* within the *Herpesviridae* family (Table 1) [1].

**Figure 1:**
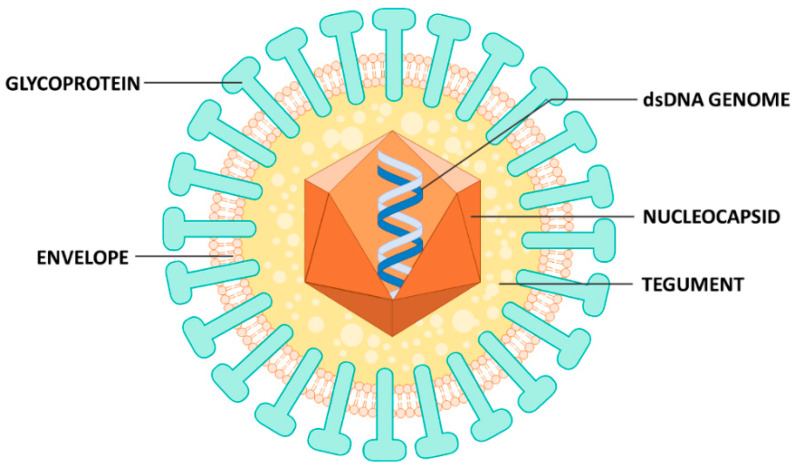
Structure of HCMV virion. Mature virions are coated by an envelope, from which viral glycoproteins protrude, and contain a double-stranded DNA genome enclosed within an icosahedral symmetry capsid, that is surrounded by tegument.

The expression of HCMV genes, similar to that of all other herpesviruses, occurs in a temporal cascade consisting of immediate-early (IE), early (E), and late (L) genes. The viral particles are formed by a double-stranded DNA (dsDNA) genome (~230 kb), an icosahedral capsid, followed by the tegument (a proteinaceous layer), and a coating known as pericapsid or envelope, which confers the virion a quasi-spherical shape (Figure 1), a feature shared with all other herpesviruses.

HCMV can infect a broad cell range that includes epithelial cells of glandular and mucous tissues, smooth muscle cells, fibroblasts, macrophages, hepatocytes, dendritic cells, and vascular endothelial cells (ECs) [2]. After primary infection, similar to other members of the herpesvirus family, HCMV can establish latency in the host that can be reversed even after many years by any number of stimuli [3,4].

A typical characteristic of HCMV, which originally granted the virus its name, is that of forming in the infected cell a voluminous intranuclear inclusion body and one or more intra-cytoplasmic inclusion bodies, the so-called “owl’s eye” inclusions, made up of clusters of newly formed viruses and lysosomes. The formation of such bodies generally results in increased cellular volume, a phenomenon defined as cytomegaly. Studies on HCMV began in the early 1900s when particular attention was paid to the owl’s eyes found in biopsies from stillborn fetuses and later in the kidneys and parathyroid gland cells of organ transplant patients [4].

HCMV efficiently spreads through infected body fluids, and it can also be transmitted vertically from the mother to the fetus through the placenta, causing congenital pathologies. Furthermore, even when the primary infection is resolved by an effective cellular immune response, a population of latently infected myeloid cells can persist in bone marrow monocyte precursors, thereby contributing to the risk of transferring HCMV along with organs and tissues following transplantation.

HCMV is a common pathogen of global clinical relevance, with worldwide seroprevalence ranging from 56% to 94% [5]. The viral spread in the global population is enormous, mainly due to the asymptomatic mode of infection, followed by a constant shedding of the virus through body fluids (e.g., milk, saliva, cervical secretions, and tears), which can last for months or even years.

HCMV is particularly dangerous for the following target categories of individuals [6]: (i) immunocompetent hosts, where it causes asymptomatic infections or a slight form of mononucleotic-like pathology; (ii) immunocompromised individuals such as patients suffering from human immunodeficiency virus (HIV) or undergoing bone or organ transplants; and (iii) congenitally infected newborns, who can be infected in utero, postnatally, or via breastfeeding. Of note, the prevalence of congenital HCMV (cHCMV) disease is much higher than that of Down syndrome, spina bifida, or fetal alcohol syndrome [7].

The host immune status ultimately determines the outcome of the infection as immunocompromised conditions predispose the patient to a primary infection or determine the reactivation of a latent one. In this regard, HCMV is notoriously famous for its ability to cause congenital anomalies and long-term neurological sequelae in newborns. Furthermore, it can also trigger the development of serious pathologies in solid organ or stem cell transplant recipients that are not always resolved by currently available antivirals, thereby leading in some cases to death [8].

This review provides a summary of the general characteristics of HCMV as well as its strain variability, dissemination, latency, reactivation, pathogenesis, prevention, and treatment.

## 2. Pathogenesis

**Figure 2:**
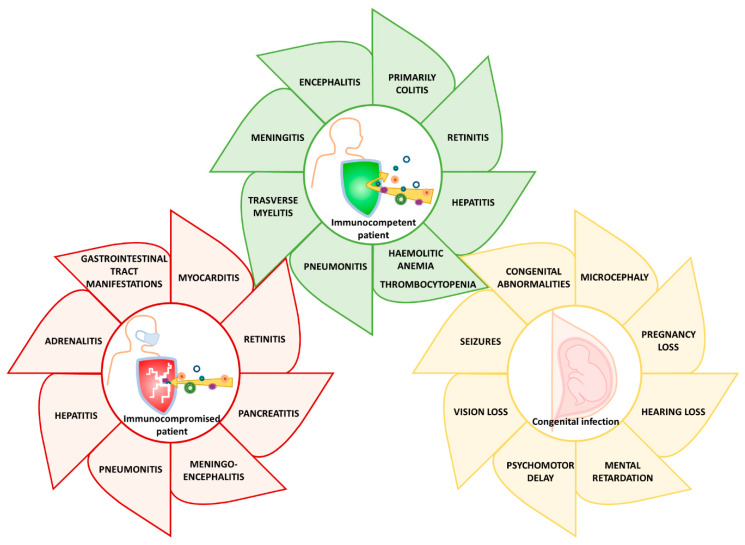
HCMV clinical manifestations in immunocompetent individuals with severe HCMV infection, in immunocompromised people, especially in acquired immune deficiency syndrome (AIDS) patients, transplant recipients, and upon congenital infection.

HCMV pathogenesis and clinical features of infection in various patient populations are summarized below (Figure 2).

### 2.1. Infection of Immunocompetent Adults

HCMV infection commonly occurs in healthy adults and children, with a prevalence gradually increasing with age [9]. When symptomatic, it results in a mononucleosis-like syndrome with a less prominent cervical lymphadenopathy than that caused by the Epstein-Barr virus (EBV) [10]. One of the symptoms is rash, which only manifests in 30% of HCMV mononucleosis cases [11]. Noteworthy, a minority of primary HCMV infections result in relapsing symptoms (i.e., fever, night sweats, fatigue, myalgia, arthralgia, and transaminitis), which can last for several weeks [10] or, less frequently, lead to multi-organ failure [12,13], even though the severe tissue-invasive disease is usually limited to critically ill or immunodeficient patients [14]. A study describing the various clinical manifestations of 290 immunocompetent patients with severe HCMV infection showed that the gastrointestinal tract is the preferential organ affected, primarily in the form of colitis, followed by morbidities of the central nervous system (CNS) (i.e., meningitis, encephalitis, and transverse myelitis), hematological abnormalities (i.e., hemolytic anemia, and thrombocytopenia), the involvement of the eye (uveitis and retinitis), liver (hepatitis), and lung (pneumonitis), and thrombosis of the arterial and venous system [15]. Although several studies have reported a rapid clinical improvement in immunocompetent patients with severe HCMV infection after anti-HCMV therapy, criteria for specific antiviral pharmacological treatments are not well established [12]. Randomized controlled trials should therefore be conducted to determine in which cases anti-HCMV therapy in immunocompetent patients with symptomatic HCMV infections is needed.

### 2.2. Infection of Immunocompromised Patients

In the immunocompetent host, HCMV and immunity coexist in a delicate balance. When the host immune system is compromised—i.e., in individuals with acquired immune deficiency syndrome (AIDS) and other immune diseases, post-transplant and intensive care unit (ICU) patients, and, to some extent, elderly people—the virus can exert its full pathogenic potential. Its reactivation in immunocompetent hosts, which occurs intermittently throughout life, triggers a lifelong IgG-mediated immunologic response that keeps in check viral replication. In contrast, uncontrolled viral replication occurs when populations of HCMV-specific CD4^+^ and CD8^+^ T cells are not well preserved, as observed in immunocompromised hosts, leading to severe clinical disease [16].

### 2.3. Cytomegalovirus and HIV

HIV-infected individuals are generally co-infected with HCMV [17]. Prior to the introduction of highly active antiretroviral therapy (HAART) in developed countries, about 40% of HIV-infected patients would suffer from severe HCMV disease [18]. Currently, durable suppression of HIV viremia has increased the overall patient life quality and expectancy and reduced to a minimum the pathologies associated with HCMV viral reactivation. Nevertheless, comorbidities remain problematic for HIV patients. In this regard, a close relationship between HCMV infection and HIV persistence has been reported. This is probably due to the fact that HIV-driven CD4^+^ T-cell loss and dysfunction may lead to HCMV replication and subsequent expansion of CD8^+^ T cells. Indeed, an elevated number of CD8^+^ T cells and a low CD4^+^/CD8^+^ T-cell ratio have been observed in individuals co-infected with both viruses but not in patients infected with HIV or HCMV alone [19]. Fittingly, Hunt et al. [20] demonstrated that CD8 T-cell activation could be reduced in HAART-treated HIV patients with incomplete CD4 T-cell recovery by administering anti-HCMV drugs, attesting that HCMV plays a significant role in immune activation in HIV patients. Moreover, persistent HCMV replication modulated longevity and proliferation of HIV-infected cells, improved the recruitment of new HIV target cells, and stimulated HIV transcription, thereby creating an HIV reservoir favoring AIDS progression. Clinically, retinitis has been shown to be the predominant pathology in AIDS patients (20–30%), and it usually appears at the late stages of the syndrome in patients with low CD4 count [21]. HCMV retinitis in HIV patients is commonly observed in two different forms: fulminant or indolent, both characterized by minimal or completely absent vitreous and anterior chamber inflammation [22]. If left untreated, HCMV infection of retinal cells may cause subacute retinal destruction, which can result in irreversible blindness. Paradoxically, HAART therapy while restoring the patient immune system can lead to a new pathology, known as immune recovery uveitis (IRU), which is equally destructive to the host tissue and deleterious to the quality of the patient’s life [23]. Following retinitis, the most prevalent clinical manifestations are the following: colitis(in the US, 5–10% of HIV patients with low CD4 lymphocyte counts were affected by enterocolitis prior to the availability of HAART therapy [24]), esophagitis (most commonly due to co-infection with either herpes simplex virus or *Candida albicans*), pneumonitis, encephalitis, hepatitis, and adrenalitis [25].

### 2.4. Cytomegalovirus and Transplant Patients

HCMV is one of the most frequently encountered opportunistic viral pathogens in transplant patients: a primary infection can occur in seronegative individuals after organ transplantation while a latent infection can be reactivated in seropositive individuals due to immunosuppressive treatment. The risks of HCMV-related complications in transplant recipients (R) vary according to the serostatus of the donor (D): HCMV D^−^/R^−^ transplantation is classified as low-risk, HCMV D^+^/R^+^ or D^−^/R^+^ as medium-risk, and HCMV D^+^/R^−^ as high-risk [26,27]. The most prevalent clinical manifestations in HCMV-transplanted patients are gastrointestinal symptoms, mainly affecting the upper digestive tract, whereas diarrhea is a rare occurrence indicative of colon involvement. Meningoencephalitis, clinical hepatitis, myocarditis, and pancreatitis are more common than respiratory symptoms, which in fact indicate more severe disease and may require admission to an ICU. Transplant patients without HCMV prophylaxis may display a spectrum of clinical manifestations that vary in severity from patient to patient, depending on additional personal illness risk factors, type of transplant procedure, the immunological match between donor and recipient, and immunosuppressive drugs being administered. For instance, patients treated with mammalian target of rapamycin (mTOR) inhibitors display a very low incidence of HCMV. The incidence of HCMV also correlates with the type of transplant: about 50% among pancreas or kidney–pancreas recipients, 50–75% in lung or heart–lung recipients, 9–23% in heart recipients, 22–29% in liver recipients, and 8–32% in kidney recipients [28,29]. Moreover, in patients undergoing allogeneic hematopoietic stem cell transplantation (HSCT), HCMV can lead to fatal infectious complications related to host immune recovery—about 30–70% of non-autologous and 5% of autologous HSCT-patients develop HCMV disease. Pneumonia is the disease most highly associated with HCMV infection of HSCT patients, frequently leading to death despite aggressive treatment with antiviral agents and adjunctive therapies [30]. The reasons for the development of different clinical sequelae among all the aforementioned types of transplanted patients is probably due to a combination of the following factors: (i) the nature of the proinflammatory cytokine cocktail arising after organ transplantation; (ii) the duration of HCMV replication—most transplant recipients display acute HCMV infection, which subsequently results in disease in a relatively short time frame, whereas, for example, congenitally infected infants and HIV patients can display high levels of HCMV replication for several months; and (iii) the status of the immune system response. For instance, HCMV pneumonitis only occurs when patients can activate their immune system [31].

Interestingly, conflicting results have been reported regarding the association between early cytomegalovirus reactivation and relapse after HSCT. Some studies suggest that HCMV replication after transplantation is associated with a decreased relapse risk [32,33,34,35,36,37], while others highlight that HCMV’s protective effect is restricted to patients with acute myeloid leukemia (AML) [38,39] and cannot be extended to patients with acute lymphoblastic leukemia (ALL) [40], lymphoma [41], myelodysplastic syndrome (MDS) [42], or observed in pediatric leukemias [43]. Furthermore, a more recent study by Peric et al. [44] reports that the protective HCMV effect has been pronounced in patients with myeloproliferative malignancies, while, at the same time, confirming the fact that such effect has not been observed in patients with lymphoproliferative disorders, in concordance with other studies [40,41]. Moreover, the same study highlights a significant reduction of relapse in patients with myeloproliferative neoplasms (MPN) associated with early CMV reactivation [44]. Finally, some evidence suggests that the beneficial effect of HCMV is mostly related to the conditioning regimen and restricted only to patients who receive myeloablative chemotherapy (MAC) before undergoing HSCT [45]. Despite the mounting evidence, the impact of HCMV reactivation on the patients’ overall survival (OS) has been largely regarded as controversial due to its known negative effect on non-relapse mortality (NRM). Thus, it remains largely unclear whether these conflicting reports can provide a more detailed insight into the distinct protective mechanism or they simply reflect other variables, including the sample size of the studied transplant groups. Therefore, larger prospective studies with a significant follow-up on all patients with different malignancies, monitored and treated in a homogeneous manner are needed to fully elucidate the underlying mechanism responsible for the exact effect of HCMV reactivation. These findings may ultimately lead to a significant improvement in patient management, donor selection strategies, or more personalized preemptive treatment of HCMV infection in posttransplant patients with particular malignancies.

### 2.5. Congenital and Neonatal Infection

HCMV is the major infectious cause of congenital abnormalities. The incidence of cHCMV infection due to primary and non-primary maternal HCMV infection is ~0.4–0.8% in developed countries. In general, the risk of transmission correlated with the stage of pregnancy is higher in later stages and lower in earlier ones, but in either case HCMV infection is generally associated with severe clinical sequelae in the fetus. In developed countries, ~40% of women in reproductive age are HCMV seronegative, 1–3% of whom may contract primary HCMV infection during pregnancy. The most vulnerable groups include adolescents, mothers, and caregivers in close contact with young children (e.g., teachers, nurses, etc.). Primary maternal HCMV infection has a 30–40% risk of transmission to the fetus. Importantly, HCMV reactivation or reinfection can also occur in women who are seropositive prior to pregnancy, but, in such cases, the rate of HCMV transmission is only ~1% [46,47].

The hypothesis that pre-existing maternal immunity may favor low HCMV transmission rates has long been debated. In this regard, Coppola et al. [48] performed a systemic review of the literature using Preferred Reporting Items for Systematic Review and Meta-Analyses (PRISMA) [49] guidelines and identified 19 studies assessing congenital HCMV birth prevalence in HCMV-seropositive mothers. All these studies reported low levels of congenital HCMV birth prevalence (0.4–0.6%) in seropositive mothers, in good agreement with previous findings by Lanzieri et al. [50], who systematically reviewed postnatal HCMV prevalence in developing countries. Moreover, 11 studies reported HCMV maternal seroprevalence and HCMV birth prevalence rates of 84–100% and 0.6–6.1%, respectively.

Even though most infants with cHCMV are asymptomatic, they may develop health problems at birth or later. In the most severe cases, cHCMV can cause the death of the unborn baby. In all other cases, the most clinically relevant signs at birth may include microcephaly, hepatosplenomegaly, retinitis, intrauterine growth restriction, seizures, rash, and jaundice. Some children with cHCMV infection may also suffer from long-term health problems, such as hearing loss, which can be present at birth or may develop later even in asymptomatic infants, developmental and motor delay, vision loss, and seizures. Cognitive impairment and retinitis have also been observed in asymptomatic children but at much lower rates compared to symptomatic children [51]. Prevention of HCMV remains elusive given the lack of drugs capable of treating HCMV infection in pregnant women. This aspect, together with the fact that maternal immunity has a protective role against cHCMV infection, suggests that vaccine development remains the most viable option to avoid HCMV vertical transmission.

## 3. Dissemination

HCMV exploits both vertical and horizontal transmission. Vertical transmission occurs through the placenta [52,53,54], during birth (with genital secretions), or postnatally through breast milk [55,56,57]. Horizontal transmission takes place via organ transplant [58,59], blood transfusion, or direct contact with contaminated body fluids, such as urine, breast milk, and genital secretions [60,61,62].

In most of these cases, because they cover all body surfaces, epithelial cells of the skin and internal mucosa are the first site of HCMV infection. For instance, infection through breastfeeding starts from the oral mucosa, moves to the gastrointestinal tract, which can support a productive infection, and eventually disseminates throughout the body. In contrast, studies using murine CMV (MCMV) have shown that, after oral/intranasal inoculation, the infection can only evolve in the upper respiratory tract but not in the gut [63]. It remains a matter of debate how HCMV disseminates from the upper respiratory tract throughout the body.

**Figure 3:**
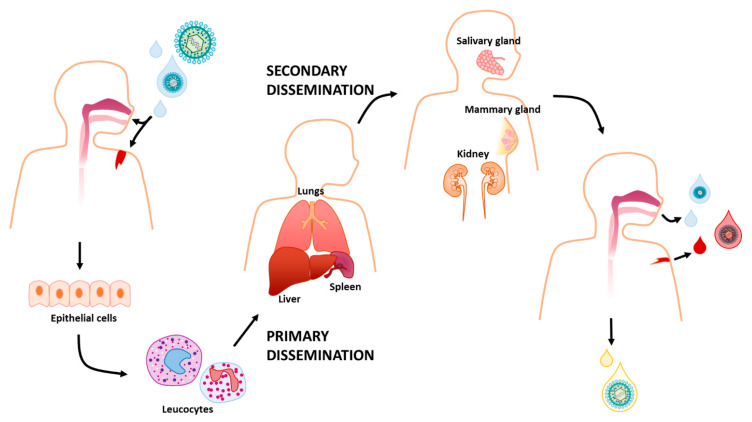
HCMV can be transmitted directly from person to person through bodily fluids including saliva, urine, cervical, and vaginal secretions, breast milk, semen, blood, and tears. It infects a new host usually by getting in through the upper gastrointestinal gastrointestinal tract or the respiratory tract. Here, the epithelial cells are often the first site of infection and from there HCMV infects leucocytes that traffic around the body. This is correlated with a process called primary viral dissemination that leads to the infection of multiple tissues, such as lungs, liver, and spleen. Afterwards, secondary viral dissemination spreads the infection to secretion-producing organs, such as salivary and mammary glands and kidneys, which shed the virus.

It is widely acknowledged that HCMV can spread systemically via leucocytes, a process associated with short-duration viremia, during which infection of the lungs, liver, and spleen occurs primarily through viral dissemination [64]. Subsequent secondary dissemination leads to the infection of salivary glands, breast, and kidneys, all secretion-producing organs that release the virus into the environment for months, even years, favoring intra-host transmission [64]. According to a model whereby primary dissemination produces many viral particles that then infect other organs generating even more virus progeny, it would be expected a gradual increase in viral burden during primary infection [64] (Figure 3).

However, animal studies using the MCMV model showed a biphasic rather than a gradual increase in viremia, suggesting a much more complex scenario [65].

Recently, Jackson and Sparer [66] demonstrated that cells of the upper respiratory tract, once infected, release not only viral progeny but also chemotactic factors. According to the proposed model, these chemokines trigger the recruitment of innate immune cells, which after being infected further spread the virus to secondary organs and body fluids [66]. Fittingly, HCMV DNA has never been found in the form of free circulating viral particles, except for highly fragmented DNA [67]. Consistent with the lack of free circulating HCMV, leukocyte-depleted blood from seropositive donors prior to blood transfusion prevents HCMV transfer [68,69], indicating that HCMV viremia is mostly cell-associated. More recently, Farrell et al. [63] showed that the first cells to be infected after nasal inoculation with MCMV are alveolar macrophages and type 2 alveolar epithelial cells. Entry into epithelial cells and macrophages occurs through endocytosis and is followed by subsequent pH-dependent fusion with the endosomal membrane, mediated by the viral envelope glycoproteins gB and gH/gL/gO and the pentameric complex formed by gH/gL/UL128, UL130 and UL131A [70,71]. Then, the local spread is thought to occur through direct cell-to-cell transmission, mediated in part by the HCMV gene *US28* [72].

The main cell types contributing to hematogenous dissemination, albeit to different extents, include polymorphonuclear cells (PMNs), monocytes, ECs, and dendritic cells. After recruitment to the first site of infection, these cells are highly prone to infection themselves, thereby becoming potential vehicles for HCMV transmission, even though most of them are unable to support a complete viral replication cycle [73,74,75,76]. Consistently, HCMV is frequently found in PMNs from immunocompromised patients [74], in which viral replication is generally abortive and non-productive [73]. The infection of PMNs most likely occurs by transient microfusion between ECs and PMNs after an initial direct contact mediated by the pentameric complex. Successively, infected PMNs transfer the virus particles to other cell types [77]. On the other hand, other studies using MCMV do not seem to support the hypothesis that neutrophils play a role in HCMV dissemination, since their depletion did not alter primary or secondary viral diffusion [78], whereas depletion of monocytes, macrophages, and NK led to reduced viral dissemination [63,79,80]. However, it is important to point out that there are substantial differences between human and murine CMV, exemplified by the lack of the MCMV CXC chemokine homolog involved in neutrophil migration [78].

HCMV carries two genes, *UL146* and *UL147*, which encode for the two chemokine homologs vCXCL-1 and vCXCL-2, respectively, involved in the recruitment of innate immune cells [81,82,83,84]. pUL128, a key component of the pentameric complex, is an important chemokine that, once released in the extracellular milieu, regulates monocyte migration [85]. Likewise, MCK2 in MCMV acts as a strong attractant of monocytes, which appear to be conserved dissemination vehicles across species [86,87]. Monocyte-driven hematogenous spread is probably the result of the close proximity of these cells to the vascular epithelium, which renders them particularly susceptible to infection with viral particles originating from productively infected ECs. Once fully differentiated into tissue macrophages [75], they can, in turn, spread the infection to the organs where they transmigrated [88,89,90].

Infected ECs also play a fundamental and active role in HCMV dissemination. In fact, HCMV infection of ECs supports viral replication and promotes the enhanced expression of the adhesion molecules ICAM-1 and vCAM-1 [91,92], as well as increased vascular permeability, which promotes recruitment of leucocytes, direct contact [92] and migration through the endothelial layer.

Dendritic cells (DCs) are antigen-presenting cells that keep in check foreign pathogens by influencing T-cell activation and differentiation in the draining lymph node through different mechanisms [93]. Immature DCs localize in all mucosal and epidermal surfaces of the body where they uptake HCMV infectious particles, thereby initiating the maturation process during their migration to the draining lymph node. Upon localization in this new site, the newly mature and permissive DCs are capable of transferring the virus to other cells [94].

In summary, there is still certainly a long way to go before we can fully understand the pathogenesis of HCMV infection, but the aforementioned mechanism of HCMV dissemination proposed by Jackson and Sparer [66] appears to be putting together many pieces of the puzzle.

## 4. Latency

Viral latency is defined as the maintenance of the viral genome without any production of infectious progeny until this dormant genome can reactivate in response to specific stimuli and initiate a productive infection. It is, therefore, becoming increasingly clear that a better understanding of latency and subsequent reactivation may be crucial to elucidate HCMV pathogenesis and develop therapeutics targeting latent virus reservoirs. This is a particularly important aspect given that all commercially available drugs for the treatment of HCMV diseases only target lytic but not latent infections.

For decades, latency was considered as a silent state of the infection, characterized by overall suppression of viral gene expression aimed at preventing the detection and activation of the immune system. However, several recent studies have shown latency to be a dynamic phase of the infection, where viral gene expression triggers a transcriptional cascade responsible for subverting host cell functions, such as cell survival, genome carriage, and immune evasion [95,96,97].

The main site where HCMV is known to establish latency is in cells of the myeloid lineage. The idea that infectious viral particles could be carried by white blood cells came from the observation that blood transfer from healthy seropositive donors to immunosuppressed seronegative recipients often resulted in HCMV disease [98,99,100], and that transfusion of leukocyte-depleted blood reduced the incidence of HCMV disease [101].

However, it was only thanks to the increased sensitivity of the PCR technique that HCMV DNA could be found in naturally latently infected peripheral blood mononuclear cells (PBMCs), in particular monocytes and CD34^+^ progenitor cells isolated from the bone marrow [102,103]. Consistent with the notion of myeloid cells being a bona fide site of latency, HCMV IE RNA expression has also recently been detected in DCs isolated from peripheral blood of healthy individuals [104].

Investigations on viral gene expression during natural infection are limited by the fact that only 0.004–0.01% of mononuclear cells from seropositive granulocyte colony-stimulating factor (G-CSF)-stimulated donors carry viral genomes, with a low copy number of 2–13 genomes per infected cell, as judged by PCR-driven in situ hybridization [105]. For this and other reasons, leukemic cell line models such as THP-1 and CD34^+^ Kasumi 3, as well as several embryonic stem cell lines, have been preferentially used as bona fide and low-cost models to study latency and reactivation in vitro [106,107,108,109].

As CD34^+^ cells are also lymphoid cell progenitors, several studies have tried to explain why myeloid cells are then the only cell lineage able to carry latent viral genome. By performing experimentally latent HCMV infection of CD34^+^ progenitor cells, Poole et al. [110] observed an increase in the cellular transcription factor GATA-2, a key regulator of myeloid differentiation, suggesting that the virus may only engage myeloid-committed cells, promoting their survival. GATA-2 is also involved in the differentiation of hematopoietic progenitors along the endothelial lineage. However, HCMV DNA could not be detected by PCR in ECs from the saphenous vein of healthy individuals, contrary to the hypothesis that microvasculature is a site of HCMV latency [111].

To establish latency, HCMV must stop the production of infectious viral particles through suppression of viral gene expression and, at the same time, induce the expression of latency-associated viral genes [112,113,114,115,116]. One of the key events required to initiate a state of latency involves the repression of the viral major immediate-early promoter (MIEP), which is sufficient to prevent the expression of E and late L genes as well. Transcriptional inactivation of this region is achieved through induction of repressive chromatin marks—e.g., histones methylation and recruitment of heterochromatin protein 1 (HP-1)—and repressive transcription factors [117]. Concurrently, differentiation of latent CD34^+^ cells or monocytes to macrophages or DCs induces re-activation of the promoter through histone acetylation and loss of HP-1, with subsequent expression of *IE* genes and re-entry into the lytic cycle [76,104,118,119], indicating that dynamic regulation of the MIEP is a first and crucial step to control latency/reactivation.

One of the most widely accepted hypotheses is that the virus gene expression upon latency is mainly characterized by a robust suppression and shut down of almost all viral genes, an expression profile similar to that of the late lytic cycle. In this regard, it has been proposed that, in latently infected cells, the timely transcriptional cascade of productive infection may be prematurely interrupted by cellular mechanisms. Alternatively, there could be, right after viral entry, early induction of viral gene expression followed by massive repression of viral transcription [120].

As mentioned above, rather than being quiescent, latent HCMV infection induces the expression of a certain amount of viral genes. The most sophisticated mechanism for modulating the host cell environment without attracting an immune response is mediated by non-immunogenic molecules, such as small RNA transcripts. Assessing both experimentally and naturally latent infected cells by next-generation sequencing, Rossetto et al. [121] identified two long non-coding (nc) RNAs (lncRNAs), RNA4.9 and RNA2.7, and mRNAs encoding replication factors UL84 and UL44. Of note, RNA lnc4.9 in concert with latently expressed *UL84* was shown to interact with members of the polycomb repressor complex 2 (PRC2), which potentially represents an additional step of silencing of the MIEP through their histone methyltransferase activity [122].

Across its genome, HCMV also encodes at least 20 viral microRNAs (miRNAs) identified first in lytically-infected cells [123], but also in latently-infected cells THP-1 by Meshesha et al. [124], using deep-sequencing analysis. More recently, two similar studies were performed using instead primary latently-infected cells that more resemble the in vivo situation, even though they showed conflicting results to some extent [125,126]. The advantage of using miRNAs, besides their non-immunogenic state, stems from their ability to modulate the expression of multiple targets involved in immune evasion, survival, and proliferation of HCMV-infected cells, as well as virus reactivation [127]. One example is the miR-UL148D that during the lytic cycle promotes T-cell chemotaxis by targeting CCL5 (RANTES), while during latency it may trigger activin signaling, thereby inhibiting pro-inflammatory cytokine secretion [126,128]. In addition, even cellular miRNAome was shown to be widely affected by HCMV latent infection [129].

A restricted amount of viral proteins is detected in naturally latent infected cells, even though their exact role in latency is only partially clear. These include: US28, a constitutively activated chemokine receptor acting as a chemokine sink [130]; viral IL-10, which can downregulate MHC II surface expression and modulate CD4^+^ T-cell recognition [131], UL144, a decoy tumor necrosis factor receptor (TNFR), which inhibits T cell proliferation in vitro [132] and subverts the TH1 immune response in a TNF ligand-independent fashion [133]; LUNA (latency unique natural antigen), a protein required for reactivation [134]; and UL138, which maintains latent infection and suppresses reactivation [115]. Interestingly, this latter is a potentially druggable target in latently infected cells as it inhibits a cellular drug transporter [134].

As latency appears to be a very complex phenomenon, reactivation is often the result of a closely intertwined crosstalk between cellular and viral signals triggering multiple pathways. Indeed, *IE* gene expression alone does not seem to be sufficient to induce the production of infectious particles [76]. In this regard, the observation that differentiation of experimentally latently infected monocytes into monocyte-derived macrophages can lead to a fully permissive phenotype [88,89] implies that the differentiation status is a critical determinant of reactivation. In addition, mounting evidence indicates that inflammation may also play a role in HCMV reactivation. For instance, virus reactivation has been observed in several progenitor cell types under a variety of inflammatory conditions [135,136,137], and HCMV disease prevalence is directly associated with highly inflammatory environments [137,138,139].

**Figure 4:**
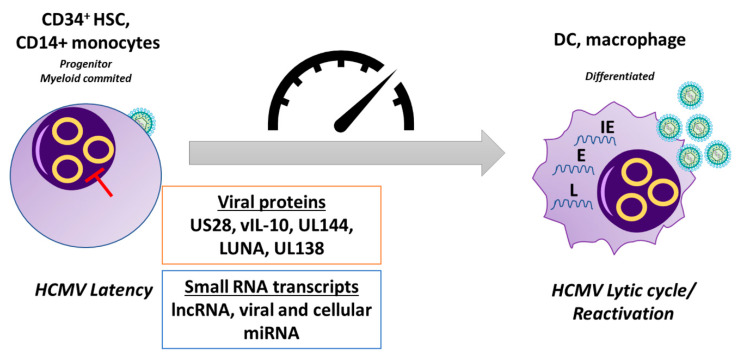
Latency. Following primary infection, HCMV can establish latency in CD34^+^ myeloid progenitor cells and is carried down the myeloid lineage. In latently-infected CD34^+^ cells and monocytes, there is a targeted suppression of lytic viral gene expression. HCMV utilizes several viral proteins and small RNA transcripts, including viral and cellular miRNAs, during latent infection to alter the signaling environment within the cell to maintain the status of latency. Differentiation of these cells to macrophages and DCs causes the derepression of the MIEP and allows initiation of the lytic transcription program, which involves a temporal cascade of viral gene transcription, allowing reactivation of de novo virus production. HCMV, human cytomegalovirus; DC, dendritic cell; HSC, hematopoietic stem cell; lncRNA, long non-coding RNA; IE, immediate-early; E, early; L, late.

In light of the above, it is becoming increasingly evident how a multiplicity of latency and reactivation pathways can determine the course of HCMV infection. These pathways appear to be independent of the clinical strains but seemingly dependent on a combination of viral and cellular factors working cooperatively to cross the threshold for reactivation of latently infected cells (Figure 4).

## 5. HCMV Strain Variation

The massive spread of HCMV infection and the wide spectrum of disease manifestations in infected patients sparked a major interest in determining the origin and mechanisms of HCMV pathogenicity. It has been 66 years since Margaret Gladys Smith isolated for the first time MCMV from salivary glands and propagated the virus in mouse cell culture—such strain is still in use and commonly called “Smith strain” [140]. One year later, she succeeded in growing submaxillary salivary-derived HCMV in human cell culture. As early as 1960, Thomas Weller isolated the so-called David strain from a liver biopsy and managed to identify serological differences between cytomegalic inclusion disease (CID) isolates [141].. Subsequently, five different HCMV clinical isolates were sequenced (FIX GenBank AC146907; TR GenBank KF021605; PH GenBank AC146904; Toledo GenBank AC146905; Merlin GenBank AY446894). The pioneering work on sequencing of the complete genome of an HCMV laboratory strain (AD169 GenBank AC146999) [142], along with numerous in vitro findings and data from several vaccine studies, revealed the existence of a substantial genetic variation among HCMV strains. In particular, the highly passaged laboratory strains AD169 and Towne (GenBank FJ616285) appeared attenuated when administered as vaccine candidates [143,144]. By contrast, the Toledo strain, which had only been passaged several times in culture, caused disease when administered to seropositive individuals [145]. Taken together, these findings suggest that the pathogenic potential of HCMV was correlated with the genetic composition of each distinct strain.

The differences among the widely used laboratory strains AD169, Towne, and Toledo were localized to multiple ORFs in the UL/b′ region of the genome, encoding viral proteins with immunomodulatory or evasive functions [145,146]. Those genes that were lost upon extensive passaging in vitro played a crucial role in promoting viral replication and immune manipulation in vivo [147]. Consistently, extensive culture passaging led to the selection of HCMV mutants lacking these genes within weeks of propagation, and it also gave rise to variations between commonly used laboratory strains [146,148,149,150].

In the following years, with the development of more sensitive sequencing techniques, such as Sanger and high-throughput sequencing [151,152], a higher number of HCMV genomes were sequenced from bacterial artificial chromosomes [153,154,155], virion DNA [156], or overlapping PCR amplicons [148]. The widespread implementation of these new techniques allowed assessing different aspects of HCMV genome variation in clinical HCMV isolates from different cohorts of infected patients, thus providing novel insights into the genetic variation upon natural infection. Up to date, the complete genomes of 351 full-length HCMV strains have been published and analyzed (National Institute of Allergy and Infectious Diseases (NIAID)-sponsored Virus Pathogen Database and Analysis Resource (ViPR) [157]) [158]. Interestingly, these sequencing data show that HCMV can be highly polymorphic among and within hosts [159,160,161], with a high level of intra-host variability comparable to that of RNA viruses [159]. Given the fact that HCMV is a large double-stranded DNA virus, a high degree of genetic variation contradicted the logical expectation that the virus would retain high genome stability [162]. This unexpected intra-host HCMV diversity was initially attributed to the rapid occurrence of de novo mutations [159,160]—i.e. new mutations occur every time the virus infects a new host, thereby giving rise to a unique viral strain for each infected individual. Eventually, HCMV infection triggers a selection event where a new genotype becomes dominant due to the selective pressure of the immune response [159]. An alternative explanation was based on the evidence that viral and host factors can contribute to the onset of HCMV genome mutations, thus fostering virus genetic drift upon infection [163,164]. However, more recent data indicate that in non-mixed infections the mutation rate of HCMV is no different from that of other DNA viruses, while HCMV acquires a high degree of variability upon mixed infections [165,166,167], extensive recombination [152,166,167,168], or reactivation of the latent virus within a single individual. Many of these genetic alterations may in turn influence HCMV cell tropism, immune evasion, and disease outcomes. Indeed, the contribution of superinfection and recombination to viral genetic variability, an intensively debated topic, could have important ramifications in viral evolution, immune adaptation, and pathogenesis, especially in congenital or transplant patients [169,170].

Substantial efforts have been undertaken by various groups to correlate infection outcomes with variation in HCMV-specific genes [145,171,172]. Even though the selection of these genes was based on data supporting their potential role in viral pathogenicity and dissemination, these studies were only limited to Sanger sequencing of polymerase chain reaction (PCR) amplicons and often focused on a small number of polymorphic (hypervariable) genes. Furthermore, in such cases, low-abundance viral populations might have been missed, and the overall viral diversity underestimated. Thus, future studies should take advantage of high-throughput sequencing for fast detection and characterization of multiple-strain infections. Ideally, as recently put forward by Davison and co-workers, the definition of HCMV natural populations should be carried out by whole genome sequencing of HCMV strains directly from clinical samples [166].

## 6. Prevention

Although the development of an HCMV vaccine had already started in the 1970s, research in HCMV vaccine discovery received a major push when in 2000 the US Institute of Medicine placed HCMV among the top priorities for vaccine development [173]. Despite these increasing efforts, an effective vaccine against HCMV is currently missing, de facto leaving high-risk populations, chiefly immunocompromised patients and immunocompetent seronegative pregnant mothers, exposed to primary infection [174]. Given that one of the main obstacles to the development of an efficient vaccine against HCMV is the lack of protection against HCMV re-infection and/or reactivation, the first objective of a newly designed HCMV vaccine should be that of shielding vulnerable populations from primary infection. The long-range goal would then be to grant permanent protection against new infections with other HCMV strains and reactivated infections, which can occur repeatedly throughout life. To reach these goals, the ideal HCMV vaccine should be able to trigger a strong humoral response, in the form of binding and neutralizing antibodies, and an HCMV-specific CD8^+^ and CD4^+^ T-cell response. With this in mind, the experimental and clinical results achieved so far predict that the ideal HCMV vaccine should include: (i) gB, promoting both humoral—primarily antibody-binding—and T-cell-mediated response [164]; (ii) pp65, triggering a potent T-cell response; and (iii) the pentameric complex (PC), which prompts a quite strong neutralizing antibody (NAb) response [175]. Indeed, PC-induced NAbs are powerful cell-to-cell spread viral inhibitors in numerous cell types, not just fibroblasts. In the following sections, we summarize the most relevant approaches for designing effective HCMV vaccines (Table 2).

### 6.1. Live HCMV Vaccines

The first attempts to develop an HCMV vaccine were focused on two attenuated strains: the AD169 and the Towne strain, both developed at the beginning of the 1970s by Elek and Stern in London and Plotkin in Philadelphia [176,177]. Their results show the vaccine to be very safe due to the absence of virus excretions—this was true even for vaccinated kidney transplant patients on chronic immunosuppression. Furthermore, these patients did not display cell-mediated immunity depression or any systemic reactions. Concerning immunogenicity, NAbs were present at levels similar to those found in the serum of patients infected with the wild type virus. Although the vaccine was able to generate both HCMV-specific CD8^+^ and CD4^+^ T-cell response, the immunity induced by vaccination did not prevent secondary infection in pregnant women, while mucosal immunity induced by natural infection did. Thus, immunization was deemed incomplete [178]. A reason for this only partial success is probably related to the fact that the two HCMV strains used to obtain the vaccines, when propagated on fibroblasts in vitro, are known to acquire mutations in *UL128L*, a subunit of the pentameric complex. In particular, the Towne strain undergoes a 2-bp insertion (TT), causing a frameshift mutation in *UL130* [154]. This event is particularly important if we consider that a functional PC is required for the virus entry into epithelial cells or ECs, whereas gB and the gH/gL dimer are sufficient for entry into fibroblasts [179]. Consistently, epithelial-neutralizing antibodies against Towne strain, harboring a mutation in PC, were 28-fold lower than those induced by natural infection [180].

To develop a live HCMV vaccine with the same safety of the Towne’s one but more protective and immunogenic, four genetic recombinants of Towne and Toledo, a low-passage HCMV strain, were subsequently generated. The chimera vaccine candidates were tested in Phase I clinical trials and found to be well tolerated and with no virus excretions in saliva or urine, with two chimeras (2 and 4) being more immunogenic than Towne [181].

In 2012, a replication-defective virus strategy was put in place for the first time. It consisted of restoring a mutated PC through serial passages of AD169-infected ECs. Subsequently, the two HCMV proteins IE1/2 and UL51 were linked to a protein domain that rendered their stability dependent on the synthetic compound Shield 1 (Shld1). That meant that HCMV could replicate normally in the presence of Shld1, whereas in the absence of such molecule viral replication was abortive due to the IE1/2 and UL51 degradation. As later confirmed in several studies, this defective virus, named V160, retained its ability to express all other AD169-specific proteins, including PC and gB, thereby eliciting both T cell and humoral responses in non-human primates as well as in humans [182]. A recently concluded Phase I study showed that sera from V160-immunized HCMV-seronegative patients have features similar in quality to those from seropositive subjects, justifying future clinical trials on this vaccine [183].

Finally, several viral vectored vaccines against HCMV have been formulated and tested in Phase I/II trials. These strategies for vaccine development employ heterologous viral vectors to deliver HCMV-encoded antigens such as gB, pp65, IE1, and, in some cases, PC proteins. While viral vectors cannot replicate completely when injected into humans, they have an optimal safety profile and can efficiently carry the desired viral antigens. The following heterologous viral vectors were tested as HCMV vaccines: canarypox virus vector, alphavirus vector (Venezuelan equine encephalitis (VEE) virus), lymphocyte choriomeningitis virus vector, modified vaccinia Ankara virus (MVA) vector, and adenovirus type 6 vector [184].

In conclusion, although consistent progress has been made over the past 45 years, and several potential candidates have emerged, an effective live HCMV vaccine has yet to be successfully developed.

### 6.2. Non-Living HCMV Vaccines

The simplest non-living HCMV vaccines are the so-called subunit vaccines, obtained by combining subunit immunogens with an adjuvant. The first one was made with the recombinant glycoprotein gB with the microfluidized adjuvant 59 (MF59) [185]. Several Phase II clinical trials in both adults and toddlers showed that after three doses given at 0, 1, and 6 months gB/MF59 was able to induce a reasonable NAb titer. Even though these studies showed that gB when injected alone as a vaccine had a certain degree of protection against HCMV infection through the mucosal route, the antibody response was short-lived and disappeared within a year [186].

A different strategy for non-living vaccines is based on genetic programming of host cells—via uptake of naked DNA or RNA—to express the desired immunogens in vivo. In this regard, a plasmid-based DNA vaccine, called the ASP0113 bivalent DNA vaccine, managed to reach Phase III clinical trials. This vaccine was obtained using two plasmids: VCL-6365, encoding the extracellular domain of gB, and VCL-6368, encoding a modified pp65 protein kinase gene [187].

Likewise, the use of self-replicating RNA to express viral proteins has made progress in recent years. Specifically, an mRNA-based multiantigenic vaccine containing gB, pp65, and PC injected in mice was capable of inducing potent cell-mediated and humoral immune responses. Although an effective cell response to pp65 was hampered by the presence of other HCMV antigens, it could be restored by sequential immunization of pp65 followed by PC + gB + pp65 [188]. In a very recent study a new RNA-based vaccine, consisting of lipid nanoparticle (LNP)-encapsulated nucleoside-modified mRNA encoding full-length gB, was tested on a group of young New Zealand White rabbits. The gB nucleoside-modified mRNA-LNP vaccines were highly immunogenic with similar kinetics and comparable peak gB-binding and functional antibody responses induced by gB/MF59 subunit vaccine. However, rabbits immunized with nucleoside-modified mRNA-LNP showed significantly longer duration of vaccine-induced antibody responses [189].

Other heavily studied approaches include the use of HCMV peptides, to elicit cellular immunity in transplant patients, and enveloped virus-like particles (VLPs) in order to stimulate wild type viruses in the absence of viral DNA, with VLPs exhibiting on their surface gB and, in some cases, PC different variations of VLPs have had some success in animal immunization tests [190,191].

One different approach involves the purification of dense bodies (DBs), including virion tegument and envelope proteins but not viral genome, produced by HCMV-infected cells. DB-injected mice did display both T-cell and NAb responses [192].

In summary, the antigens needed for successful vaccination against HCMV are well known from the literature. Thus, further effort will be required in combining such antigens to achieve a durable response that would protect an individual for an extended time.

## 7. Treatment

The high incidence and clinical manifestations of HCMV infection underscore the need for efficient antiviral therapies in treating disease in immunocompromised patients and congenitally infected infants.

Four compounds are currently approved for systemic treatment or prophylaxis of HCMV infections: ganciclovir (GCV) and its oral prodrug valganciclovir (VGCV), cidofovir (CDV), foscarnet (FOS), and, more recently, letermovir (LTV) [193,194] (Table 3).

Among the latter, GCV, CDV, and FOS exhibit similar activities, as they target viral DNA polymerases, consequently inhibiting the synthesis of HCMV DNA [195]. In contrast, LTV blocks DNA packaging in the viral capsid by interfering with the viral terminase complex [196]. Furthermore, in the late 1990s, a fifth antiviral compound, fomivirsen, also known as Vitravene, was approved for the treatment of HCMV infection by the Food and Drug Administration (FDA) and the European Agency for the Evaluation of Medicinal Products (EMEA). Fomivirsen, an antisense 21-mer phosphorothioate oligonucleotide (5′-GCGTTTGCTCTTCTTCTTGCG-3′) complementary to the mRNA encoding the IE-2 protein, is used to treat HCMV-induced retinopathy in immunocompromised subjects [197]. Prophylactic treatment with acyclovir has also been approved in some countries, although the effectiveness of this approach is moderate.

The front-line therapy for HCMV infections is GCV, which was approved for medical use in 1988 and is now routinely used to treat congenitally HCMV-infected infants [198] and immunocompromised hosts with HCMV disease [199] or given as a prophylactic agent to prevent HCMV disease [194,200,201]. GCV, a monosodium salt of 9-(1,3-dihydroxy-2-propoxymethyl)-guanine, is an acyclic nucleoside analog administered by intravenous infusion that, when phosphorylated to form GCV triphosphate (GCV-TP), inhibits the viral UL54 DNA polymerase and, by competing with deoxyguanosine (dGTP), curbs the elongation of viral DNA. GCV is administered in an inactive form and is selectively phosphorylated in the infected cells by the HCMV UL97 kinase [202]. This kinase is responsible for the conversion of GCV to GCV monophosphate (GCV-MP), while the subsequent phosphorylation steps generating the active form of the drug (GCV-TP) are controlled by cellular kinases [203]. However, its moderate antiviral activity and dose-limiting toxicity hinder its efficacy and may lead to the development of drug-resistant infections, mainly in immunocompromised patients [204]. Specifically, GCV-induced cytotoxicity may result in thrombocytopenia and neutropenia in transplant recipients [205].

Secondary therapies for HCMV infections consist of CDV and FOS, which can both cause nephrotoxicity and give rise to resistant infections [193,194].

CDV was approved in 1996 for the treatment of HCMV retinitis in AIDS patients and is available only intravenously. Cidofovir, 1-[(S)-3-hydroxy-2-(phosphonomethoxy) propyl]cytosine, is an acyclic phosphonate nucleoside (ANP) acting as an analog of CMP (cytidine monophosphate). CDV presents a broad spectrum of antiviral activity; in particular, it acts against most DNA viruses (e.g., herpesviruses, adenovirus, polyomavirus, and orthopoxvirus) [206]. Since CDV is a monophosphate analog, it does not require the initial activating phosphorylation by the UL97 viral kinase. Nevertheless, to be active, CDV needs to be diphosphorylated by the pyruvate kinase, creatine kinase and nucleoside diphosphate kinase, all present at high levels in infected HCMV cells. Once activated, CDV competes with deoxycytidine triphosphate and is incorporated in the DNA as an alternative substrate of the viral DNA polymerase where it acts as a non-mandatory chain terminator [206]. CDV resistance is only associated with viral DNA polymerase mutations and occurs at a frequency comparable to that of GCV [206].

FOS was approved for the treatment of HCMV infections in 1991. Similar to CDV, FOS inhibits the replication of several types of DNA viruses (e.g., several herpes family viruses, hepatitis B virus, and HIV), but it is primarily used for the treatment of HCMV retinitis [207]. FOS is a synthetic organic analog of inorganic pyrophosphate, which reversibly inhibits the activity of the UL54 DNA polymerase. FOS resistance during long-term therapy occurs at similar rates to those displayed by CDV and GCV. However, FOS can be particularly useful against some GCV-resistant infections as the frequency of GCV cross-resistance is much lower than that observed for CDV and GCV, even though renal toxicity may limit its usefulness [208]. Combination therapy with GCV has also been investigated, but it does not seem to be more efficient than GCV alone. Despite the above limitations, the broad spectrum of antiviral activity of FOS makes this compound useful for the treatment of some GCV-resistant infections. Of note, FOS has also been approved for the treatment of ACV-resistant HSV infections. Lastly, because this agent crosses the blood-brain barrier, it is indicated for the treatment of viral infections involving the central nervous system.

Drug resistance results from the development of single or multiple mutations leading to different levels of resistance that can reduce the efficacy of the antiviral treatments. Since all these drugs, except of fomivirsen, target, directly or indirectly, viral DNA polymerase, the emergence of drug-resistant HCMV strains, often due to mutations in UL97 and/or UL54, has increasingly hampered disease management [209]. Therefore, there is an urgent medical need for new anti-HCMV agents with new mechanisms of action and fewer side effects.

Currently, a promising new class of riboside analogs with strong and specific antiviral activity against HCMV is being tested in a clinical trial. One of these drugs is maribavir, a benzimidazole riboside characterized by a novel mechanism of action based on its ability to inhibit UL97, a viral enzyme that blocks nuclear egress of viral capsids, and to interfere with viral DNA synthesis. Even though clinical trials with maribavir have not yet been conclusive, the drug is still being evaluated as a potential preventive treatment of HCMV infection [210,211].

BAY 38-4766, also called tomeglovir, is a non-nucleoside antiviral. The antiviral activity of BAY 38-4766 is due to its ability to hinder DNA maturation most likely by targeting the UL89 and UL56 genes encoding for the subunits of a multiprotein complex involved in HCMV termination [212]. Interestingly, in a guinea pig model, after infection and treatment, measurable amounts of the compound were detected in fetal blood, attesting that the drug is capable of crossing the placenta in pregnant animals [213]. BAY 38-4766 is in clinical development and has shown a positive safety profile in healthy male volunteers at single oral doses of up to 2000 mg. However, no recent studies have highlighted the current state of clinical development of this drug or related compounds in the series [213].

GW275175X is a new benzimidazole riboside class of HCMV inhibitors that can block the maturational cleavage of high molecular weight HCMV DNA by interacting with pUL56 and pUL89, the two subunits of the HCMV terminase complex [214]. GW275175X was advanced to Phase I clinical trial with an increasing dose of safety, pharmacokinetics, and tolerability, but was later set aside to prioritize maribavir testing. The clinical potential of this antiviral still requires further study.

Brincidofovir (CMX001), a prodrug of CDV, is an oral lipid-drug conjugate quickly absorbed into cells whereupon the lipid side chain is cleaved, releasing CDV to be further phosphorylated by intracellular kinases to CDV diphosphate. Since brincidofovir is not a substrate for the oxyanion transporter in the kidney, kidney damage should be reduced. Nonetheless, CDV may lead to gastrointestinal toxicity, which is quite often dose-limiting [215].

In 2017, LTV became the latest FDA-approved drug for prophylaxis of HCMV infections in allogeneic HSCT recipients [216]. LTV is a non-nucleoside 3,4-dihydroquinazolinyl acetic acid, which can be administered orally or intravenously infused. LTV acts as an inhibitor of the HCMV DNA terminase complex by interacting with the pUL56 subunit of such complex and preventing the cleavage of concatemeric DNA into monomeric genome length DNA, which ultimately inhibits DNA packaging into the virion. LVT displays good efficacy against different clinical isolates of HCMV, including GCV-resistant strains, and is ineffective against all other herpesviruses [217].

To sum up, evidence from the literature indicates that currently available anti-HCMV drugs, despite being able to interfere with viral replication through different mechanisms of action, are often associated with multiple side effects such as drug toxicity, poor oral bioavailability, and drug resistance. Therefore, innovative compounds targeting new virus components with fewer adverse effects are urgently needed to improve patient outcomes.

## 8. Conclusions

HCMV is the leading cause of congenital infections resulting in severe morbidity and mortality among newborns worldwide. This virus is highly polymorphic, particularly in genes contributing to immune modulation. Despite a large amount of HCMV research over the past few decades, the mechanisms and virulence factors contributing to HCMV pathogenesis and particular clinical outcomes remain unclear. To make matters worse, to date, no safe vaccines against HCMV infections exist and current antiviral therapies are quite unsatisfactory due to the frequent occurrence of drug resistance and toxicity.

Overall, the lack of advances in the treatment of HCMV-driven diseases clearly clashes with the widespread notion that HCMV poses “a greater threat to infants than other viruses”. Thus, an in-depth understanding of HCMV-host interactions, especially at the individual level, will be instrumental to develop new diagnostic/therapeutic tools for the clinical management of this viral disease.

## Figures and Tables

**Table 1:** Classification of human herpesviruses.

Subfamily	Genus	Species	Tropism	Global Prevalence (%)
**Alphaherpesvirinae**	Simplexvirus Varicellovirus	Human herpesvirus 1 (HHV-1)/Herpes simplex virus type 1 (HSV-1) Human herpesvirus 2 (HHV-2)/Herpes simplex virus type 2 (HSV-2) Human herpesvirus 3 (HHV-3)/Varicella zoster virus (VZV)	Mucoepithelial cells (mainly oro-facial tract), neurons Mucoepithelial cells (mainly genital tract), neurons Mucoepithelial cells, T cells, neurons	40–90 10–60 50–95
**Betaherpesvirinae**	Cytomegalovirus Roseolovirus	Human herpesvirus 5 (HHV-5)/Human cytomegalovirus (HCMV) Human herpesvirus 6A (HHV-6A) Human herpesvirus 6B (HHV-6B) Human herpesvirus 7 (HHV-7)	Epithelial cells, monocytes, lymphocytes, fibroblasts, and more Epithelial cells, T cells, fibroblasts Epithelial cells, T cells, fibroblasts Epithelial cells, T cells, fibroblasts	56–94 60–100 40–100 44–98
**Gammaherpesvirinae**	Lymphocryptovirus Rhadinovirus	Human herpesvirus 4 (HHV-4)/Epstein-Barr virus (EBV) Human herpesvirus 8 (HHV-8)/Kaposi’s sarcoma associated herpesvirus (KSHV)	Mucoepithelial cells, B cells Lymphocytes	80–100 6–50

**Table 2:** HCMV Vaccines. NAb, Neutralizing Antibody; HELF, human embryonic lung fibroblasts; PC, pentameric complex; VLPs, virus like particles.

*Live HCMV Vaccine*	Description	Clinical Trials
*Towne vaccine*	HCMV attenuated strain.	Phase I/II clinical studies evidences: (a) no virus excretion; (b) no virus latency; (c) NAb induction; (d) generation of both HCM-specific CD4 and CD8 T-cell; (e) partial protection against a secondary infection.
*Towne-Toledo chimera vaccines*	Genetic recombinant Towne and Toledo.	In Phase I clinical trials they were well tolerated and with no virus excretion. One chimera was more immunogenic than Towne.
*AD169 vaccine*	HCMV attenuated strain.	Patients did not to display cell-mediated immunity depression or any systemic reactions.
*V160 vaccine*	Replication-defective virus vaccine based on strain AD169.	Phase I study showed that V160-immunized HCMV-seronegative patients have features comparable in quality to those from seropositive subjects.
*Viral vectored vaccines*	Heterologous viral vectors used to deliver HCMV-encoded antigens: (a) canarypox virus vector; (b) alphavirus vector [Venezuelan equine encephalitis (VEE) virus]; (c) lymphocyte choriomeningitis virus vector; (d) modified vaccinia Ankara virus (MVA) vector; (e) adenovirus type 6 vector.	Many of them were tested in Phase I/II trials. While viral vectors cannot replicate completely when injected into humans, they have an optimal safety profile.
***Non-living HCMV Vaccines***		
*gB subunit vaccines*	Combination of the recombinant glycoprotein gB with an adjuvant.	Many Phase II studies showed that gB/MF59 vaccine had a certain degree of protection against HCMV infection through the mucosal route, but the antibody response was short-lived and disappeared within a year.
*DNA based vaccines*	Single or mixed combination of plasmids encoding viral antigens such as pp65, gB and IE1.	The most promising DNA-based plasmid vaccine, called ASP0113 divalent DNA vaccine, is currently in Phase III clinical trials.
*RNA based vaccines*	Different strategies: (a) synthetic self-amplifying mRNA expressing a pp65-IE1 construct and gB; (b) mRNA-based multiantigenic vaccine including pp65, gB and PC; (c) lipid nanoparticle (LNP)-encapsulated nucleoside-modified mRNA encoding full-length gB.	Currently they have been all tested only on animal models.
*VLPs vaccines*	Enveloped virus-like particles (VLPs) which exhibit on their surface gB and, in some cases, PC.	Different variations of VLPs have shown some success in animal immunization tests.
*Dense body vaccines*	Dense bodies purified from HCMV infected cell.	DB-injected mice did display both T-cell and NAb responses

**Table 3:** Antiviral agents approved for treatment or prevention of HCMV infections.

Agent	Compound Information	Viral Target	Mechanism of Action	Route of Administration	Dosage	Toxicities
Ganciclovir (Cytovene^®^)	Acyclic nucleoside analogue of guanine	UL54	When phosphorylated to Ganciclovir triphosphate inhibits the viral DNA polymerase UL54	Intravenous Oral	Induction: 5 mg/kg every 12 hours for 7–14 days Maintenance: 5 mg/kg for 100–120 days after transplant FDA approved for maintenance therapy only using 1 mg three times a day	Neutropenia Thrombocytopenia Neurotoxicity
Valganciclovir (Valcyte^®^)	L-Valyl ester of Ganciclovir	UL54	Converted to Ganciclovir in intestine and liver, inhibits the viral DNA polymerase	Oral	Induction: 900 mg twice a day for 21 days Maintenance: 900 mg once per day	Granulocytopenia Anemia Thrombocytopenia
Cidofovir (Vistide^®^)	Deoxycytidine acyclic nucleotide phosphonate analog	UL54	When phosphorylated to Cidofovir biphosphate inhibits the viral DNA polymerase	Intravenous	Induction: 5 mg/kg weekly for 2 weeks Maintenance: 5 mg/kg every 2 weeks	Nephrotoxicity Metabolic acidosis Ocular hypotony
Foscarnet (Foscavir^®^)	Synthetic organic analogue of inorganic pyrophosphate	UL54	Inhibits activity of pyrophosphate binding site on viral DNA polymerase UL54	Intravenous	Induction: 60 mg/kg every 8 hours for 14–21 days Maintenance: 90–120 mg/kg everyday	Nephrotoxicity Hypocalcemia Electrolytes imbalance Genital ulceration
Letermovir (Prevymis^®^)	Non-nucleoside, 3,4-dihydroquinazolinyl acetic	pUL56	Binds pUL56 subunit of the HCMV terminase complex preventing the cleavage of concatemeric DNA	Intravenous Oral	Prophylaxis: 480 mg once a day, through 100 days post-transplant	
